# Epidemics, Ebola and the dignity of goodbye: how public health could respect culture

**DOI:** 10.3389/ijph.2026.1610075

**Published:** 2026-07-22

**Authors:** Janet Michel, Ntuli A. Kapologwe, Marcel Tanner

**Affiliations:** 1 Global Health Mentorships, Bern, Switzerland; 2 The East, Central and Southern Africa Health Community (ECSA-HC), Arusha, Tanzania; 3 Swiss Tropical and Public Health Institute (Swiss TPH), Basel, Switzerland; 4 Department of Science, University of Basel, Basel, Switzerland

**Keywords:** culture, death as communal, Ebola epidemic, ritual, spiritual and sacred

## Introduction

The Ebola epidemic in East and Central Africa is not only a medical emergency but also a crisis of grief, dignity, and human connection. As governments struggle to contain one of the deadliest outbreaks caused by the Bundibugyo virus, with currently no specific treatment nor approved vaccine, the global health community faces unprecedented challenges in safeguarding lives. Affected communities face an impossible choice: protect themselves from infection or honour their loved ones according to deeply rooted cultural traditions [1]. Community engagement is not a peripheral aspect of epidemic response but central to whether public health measures succeed or fail. Epidemics unfold not only through biological transmission, but also through fear, grief, trust, and social relationships. The current Democratic Republic of Congo (DRC) situation and the recurring Ebola outbreaks present an opportunity to confront the long-neglected role of community and key stakeholder engagement in public health [2]. Too often, engagement has been reduced to informing or consulting communities long after decisions have already been made [3].

Since 1976, Ebola outbreaks have repeatedly been contained when proven public health measures were implemented quickly and consistently. While investments in the development of new vaccines and therapeutics are important and commendable, we must not forget that we already possess tried-and-tested tools that have worked in the past. Even if a vaccine were to be found today, without trust and acceptance of the community, vaccine hesitancy will undermine response efforts [4].

### Effective, tried and tested public health tools

Early diagnosis, rapid identification and isolation of cases, effective contact tracing, surveillance and quarantine remain cornerstone strategies for controlling outbreaks [5, 6]. These basic measures, combined with meaningful risk communication and community engagement, can effectively stop transmission. Amid broken trust and fear, it is paramount to work alongside communities in ways that respect local cultures, traditions and concerns. Effective, sustainable epidemic control and medical interventions should be built on trust, partnerships and consistent application of public health strategies that have proven to work for decades [6].

For many African societies, death is not a private event. It is communal, ritualistic, spiritual, and sacred. Funeral rites are acts of love and responsibility that connect families to ancestors, faith and identity. Janet Michel explains that in African cultures, death unites communities as relatives travel long distances to mourn, comfort families, and participate in rituals that help both the living and the dead transition peacefully [1]. During infectious disease outbreaks such as Ebola, these traditions can become dangerous. Ebola spreads through direct contact with infected bodily fluids, including those of the deceased. Traditional practices such as washing, dressing, touching, or kissing the dead significantly increase transmission. To contain epidemics, public health officials tend to respond swiftly through the so-called dignified burials, often with little cultural and traditional sensitivity. Bodies are removed rapidly by teams in protective suits with number restrictions going against traditions. Families are denied physical contact with loved ones and traditional funerals are restricted or banned. During the Liberia 2014 Ebola outbreak, mandatory cremations were introduced despite strong cultural objections. These policies may be medically justified, but overlooked that people need the dignity of farewell [7]. Top-down interventions which ignore local customs are counterproductive.

### Fear, mistrust, and resistance

The result is fear, mistrust, resistance and breach of public health acts. Mistrust leads to negative health outcomes, increases belief in conspiracy theories, vaccine hesitancy and non-compliance with public health measures [8]. Some families hid deaths, conducted secret burials for fear their relatives would disappear without a respect ceremony. Communities attacked health workers and burial teams, not because they rejected science, but because they felt excluded, humiliated, and unheard [1]. The torching of Ebola camps in DRC are a case in point. 28 infected Ebola patients fled, risking the spread of infection [9].

An estimated 20% of Ebola infections occur during burial ceremonies. The WHO’s “Safe and Dignified Burial” protocol is a starting point. Instead of excluding families, the protocol encourages their safe participation [10]. Religious leaders should be engaged with, so Muslim and Christian rites are adapted rather than abolished. Families should be allowed to observe burials, say prayers, help dig graves, choose burial garments, and participate without direct contact with the body.

The lesson from Ebola is clear: public health cannot succeed by working against culture. Public health must work with culture. As the epidemic evolves, more humane, culturally sensitive approaches that preserve emotional and spiritual core of mourning while protecting communities from infection are needed. The issue is never whether traditions should disappear entirely, but whether they could evolve safely during an epidemic [1]. Culture always wins when health measures directly conflict with deep cultural practices. Grief is not simply emotional but cultural and collective [1]. Rituals help people process trauma, maintain dignity, and preserve social bonds. Denying communities these rituals, can deepen suffering long after an epidemic ends.

### Risk communication and community engagement

Future epidemic responses must therefore prioritize adaptation of cultural practices rather than prohibit them. Community engagement should be initiated from the beginning and communities and culture should not be treated as obstacles to containment. Public health officials must not only ask, “How do we stop transmission?” but also ask the question, “How do we allow people to mourn safely?”

To achieve impact, the first step is to identify those affected and those with the potential to affect the program and engage with them beyond informing and consultation, to involvement with the aim to reach collaboration and empowerment [2]. Anthropologists, sociologists, and local community leaders should stand beside epidemiologists and doctors in designing culturally adapted outbreak responses. A humane response recognizes several truths at once, that Ebola is deadly, and grief is real and, in some cultures, sacred. Protecting populations should never strip people of their humanity and communities should never be forced to choose between safety and culture [1]. The Ebola outbreak is teaching the world that disease control rooted in fear and force consequently breeds resistance [9].

### Signals of desperation and dissonance

**FIGURE 1:**
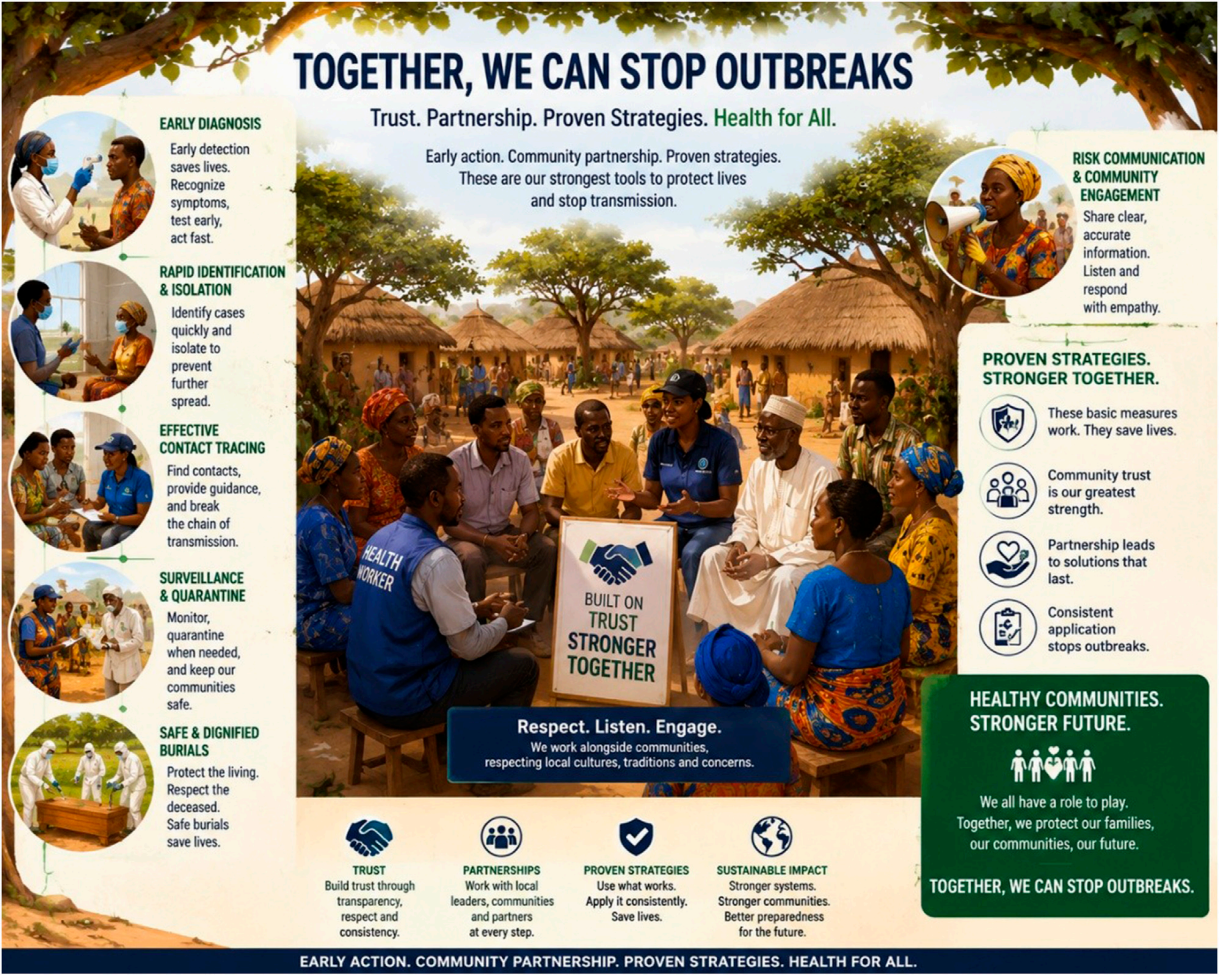
The Bedrock of Public Health Success (Bern, Switzerland. 2026).

Communities should be viewed as essential partners in designing responses that are safe, culturally acceptable, and socially legitimate. Community engagement is both an art and a science that requires systems thinking. The right to say goodbye is not a luxury during an epidemic, but an essential part of healing and human dignity. The torching of Ebola treatment camps reflects more than misinformation or resistance but signals desperation and dissonance between public health approaches, communication strategies, and cultural realities. Ituri treatment facilities were attacked after families were denied access to bodies of loved ones for traditional funerary rites, illustrating how deeply burial practices are tied to dignity, mourning, and social cohesion. See Figure 1 below.

### Conclusion

The legacy of COVID-19 has further deepened mistrust [11]. Globally, people remember relatives dying in isolation, unable to say goodbye. Grief during the pandemic has been associated with psychological and social harms when mourning practices are disrupted and when death is stripped of relational and cultural meaning [1]. Ebola responses can and should be handled differently. Approaches that recognize infection control and cultural respect are not mutually exclusive. These can be pursued together when communities are treated as equal partners rather than passive subjects of intervention [6, 7].
